# Study on the mechanism of lupenone for treating type 2 diabetes by integrating pharmacological evaluation and network pharmacology

**DOI:** 10.1080/13880209.2022.2067568

**Published:** 2022-05-28

**Authors:** Feng Xu, Mei Zhang, Hongmei Wu, Yuanmin Wang, Ye Yang, Xiangpei Wang

**Affiliations:** a College of Pharmacy, Guizhou University of Traditional Chinese Medicine, Guiyang, PR China; b College of Pharmacy, Guizhou Minzu University, Guiyang, PR China

**Keywords:** Insulin resistance, blood lipids, oxidative stress, peroxisome proliferator-activated receptor gamma, insulin receptor, insulin receptor substrate 1, insulin receptor substrate 2, glucose transporter type 4

## Abstract

**Context:**

Lupenone (LUP) is the active ingredient of *Musa basjoo* Sieb. et Zucc. (Musaceae) with antidiabetes effects, but an unclear underlying mechanism of action.

**Objective:**

Animal experiments combined with network pharmacology were used to explore the mechanism of LUP for treating diabetes.

**Materials and methods:**

Insulin resistance (IR) in male Sprague-Dawley rats with type 2 diabetic was induced using a high-fat diet and streptozotocin. The selected rats were divided into normal group, model group, positive group and LUP (2.0, 4.0 and 8.0 mg/kg) groups, and orally administrated twice daily with Tween 80, rosiglitazone or LUP. Fasting blood glucose (FBG), oxidative stress index, blood lipids and IR-related targets were detected. A network pharmacology analysis was performed.

**Results:**

Compared to the model group, LUP (8.0 mg/kg) significantly decreased the levels of FBG (22.3%), LEP (9.5%), HbA1c (14.9%) and MDA (12.3%), increased the ADPN (24.2%) levels and GSH-PX activity (12.4%) (*p* < 0.05), improved oxidative stress, lipid metabolism disorders and pancreas pathological changes, increased the mRNA and protein expression of InsR (3.7-fold and 1.3-fold), IRS-1 (3-fold and 2-fold), IRS-2 (2-fold and 1.6-fold), GLUT-4 (2-fold and 2.4-fold) in skeletal muscle and IRS-1 (6-fold and 1.6-fold), IRS-2 (5.8-fold and 1.5-fold), GLUT-4 (2.5-fold and 1.7-fold) and PPAR-γ (7-fold and 1.4-fold) in adipose tissue (*p* < 0.05). Network pharmacology analysis revealed that LUP improves IR by multiple targets and signal pathways.

**Conclusions:**

The mechanism of LUP for treating diabetes is related to improving IR. LUP has the potential to be developed as a new drug for treating type 2 diabetes.

## Introduction

Diabetes mellitus is a chronic disease characterized by hyperglycaemia (Ogurtsova et al. 2017). There are three main types of diabetes including type 1 diabetes, type 2 diabetes (T2DM) and gestational diabetes. Among these, T2DM is the most common. T2DM is a burden on global health-care systems (Radziuk and Pye 2001; Peiró et al. 2017). Clinical drugs currently used for the treatment of T2DM have many limitations and may result in adverse reactions, such as gastrointestinal symptoms, hypoglycaemia, oedema, osteoporosis and lactic acidosis (Donath 2014). Therefore, it is necessary to find an ideal drug for the safe and effective prevention and treatment of T2DM. Insulin resistance (IR) and its complications are one of the main factors leading to T2DM (Guo et al. 2021). IR refers to a decrease in the response of the biological target tissues, mainly in the liver, muscle and adipose tissue to normal physiological concentrations of insulin. Modern research finds that improving IR is an effective way to treat T2DM. Traditional Chinese medicine could treat T2DM through improving the IR, especially by increasing the sensitivity of the muscle, adipose tissue and liver to insulin (Tang et al. 2011; Sheng et al. 2014; Li et al. 2016). *Rhizoma musae* is the dried rhizome musae of the *Musa basjoo* Sieb. et Zucc. (Musaceae), a traditional Chinese medicine. Our research team has confirmed that the *Rhizoma musae*, has a good antidiabetic effect, lupenone (LUP) isolated from the *Rhizoma musae*, has a good inhibitory effect on protein tyrosine phosphatase 1B (PTP1B) and α-glucosidase *in vitro* (Na et al. 2009; Wang et al. 2012; Seong et al. 2016). Furthermore, LUP could decrease the fasting blood glucose (FBG) in mice with diabetes and improve the oral glucose tolerance and insulin tolerance in rats with T2DM (Xu et al. 2014; Wu et al. 2015, 2017). These results indicate that LUP has definite antidiabetic effects. However, the mechanism of LUP in the treatment of T2DM has not been elucidated. The current study elucidates the mechanism of action of LUP to improve IR using animal experiments and network pharmacology methods.

## Materials and methods

### Reagents

LUP was isolated from the *Rhizoma musae* by our laboratory. *Rhizoma musae* was collected from Guizhou Province, and was identified as the rhizome musae of *Musa basjoo* Sieb. et Zucc. by Professor Xiangpei Wang of Guizhou Minzu University. A voucher specimen is kept in the Drug Analysis Laboratory of Guizhou University of Traditional Chinese Medicine. *Rhizoma musae* was soak with ethanol after crushing, recovering the ethanol after filtration and concentrated to the extract, then dispersed with an appropriate amount of water and extracted with petroleum ether, ethyl acetate and *n*-butanol. Further, the resulting ethyl acetate extract was separated by silica gel column, and further purified by silica gel column and identified by comparison of its spectral data (mp, MS, ^1^H NMR, ^13^C NMR) with the reference standards. The purity of isolated LUP is over 98% by HPLC analysis as our previous report (Wang et al. 2012; Xu et al. 2014; Wu et al. 2021). Streptozotocin (STZ) was purchased from Sigma-Aldrich (St. Louis, MO). All other chemicals used in the study were of analytical grade.

### Animals

All experiments were performed on 6-week-old male Sprague-Dawley rats (200 ± 20 g) obtained from the Chongqing Tengxin Biological Technology Co., Ltd. (Chongqing, PR China). The colony was maintained under controlled conditions of temperature 23 ± 2 °C humidity, 50 ± 5%, and 12 h light/dark cycle. All the animals in the study were cared for and treated humanely according to the National Legislations of China, as well as the local guidelines. The animal experiments were approved by the Guizhou University of Traditional Chinese Medicine and the Ethics Committee for Animal Experiments of West China Hospital of Sichuan University (under registration: 2013029A).

### Induction of type 2 diabetic rats and treatment

After acclimatization for one week, the rats were randomly divided into normal group and high-fat diet (HFD) group. The rats in the normal group were fed with a standard diet *ad libitum* and the rats in the HFD group were fed with an HFD (15% cholesterol, 15% coconut oil, 15% granulated sugar and 55% standard diet *ad libitum*). After feeding for four weeks, all rats were fasted for 12 h, then the HFD group rats were injected twice with low-dose STZ (first with 30 mg/kg, i.p.; second with 28 mg/kg, i.p.; 24 h later) to induce partial insulin deficiency, and the normal group were intraperitoneally injected with sodium citrate buffer. Seventy-two hours after injection, the FBG levels of the rats were measured, and the rats with FBG of ≥11.1 mmol/L were categorized as those with T2DM. Furthermore, rats with T2DM were randomly divided into T2DM group, positive group and three LUP treatment groups. The normal and T2DM groups received the vehicle treatment (0.03% Tween 80; twice daily), the positive group was administered the rosiglitazone (1.00 mg/kg; twice daily) and the three LUP treatment groups were administered with 2.0, 4.0 and 8.0 mg/kg LUP (twice daily). After 2 weeks of treatment, six rats from each group were sacrificed by cervical dislocation, and the skeletal muscle and adipose tissues were preserved at −80 °C until further use for quantitative real-time polymerase chain reaction (PCR) test. After 4 weeks of treatment, the rest rats fasted overnight, anaesthesia was administered, and blood samples were collected. The blood samples were centrifuged at 3500 rpm for 10 min, and the serum was collected for biochemical analyses. The remaining rats were sacrificed by cervical dislocation, the pancreas was fixed with 10% neutral buffered formalin for the histological analysis and the liver tissues were fixed with Carnoy’s fixative solution to perform periodic acid-Schiff (PAS) staining, and the skeletal muscle and adipose tissues were preserved at −80 °C until further use for testing protein expression. No death was recorded and no rats were dropped out of the analysis.

### Determination of body weight and FBG

After the rats started treatment, body weight of rats from all the groups was measured every three days. All rats fasted for 12 h every weekend to measure the FBG levels using the One-touch Ultra Blood Glucose Monitoring System (Johnson & Johnson Medical (China) Ltd., Shanghai, PR China).

### Biochemical parameter analysis

The levels of serum leptin (LEP), haemoglobin A1c (HbA1c), superoxide dismutase (SOD), malondialdehyde (MDA), glutathione peroxidase (GSH-PX), adiponectin (ADPN) and free fatty acid (FFA) in serum were analysed using commercial enzyme-linked immunosorbent assay (ELISA) kit, according to the manufacturer’s instructions (Abcam, Cambridge, UK). The levels of total cholesterol (TC), triglyceride (TG), high-density lipoprotein-cholesterol (HDL-C) and low-density lipoprotein-cholesterol (LDL-C) were detected using automatic biochemical analyser (Olympus AU640, Tokyo, Japan).

### Periodic acid-Schiff staining

Distribution of glycogen particles in thin sections of liver samples was investigated using PAS staining methods. Briefly, the liver was routinely processed and embedded in paraffin. Sections of 5 μm thickness were cut. The sections of the liver tissue were deparaffinized and hydrated, immersed in 0.5% periodic acid solution for 10 min, and rinsed in distilled water for another 5 min. This was done twice. The sections were then placed in the Schiff reagent for 10 min and washed in water for 5 min before counterstained with haematoxylin for 2 min. Stained reddish dots or threadlike structures in the liver cells were considered as a positive result.

### Histological analysis

The pancreas was fixed in 10% neutral buffered formalin for 48 h. Samples were subsequently routinely processed and embedded in paraffin. Sections of 5 μm thickness were cut and stained with haematoxylin and eosin (HE) for general histopathological examination under a light microscope, counting the number of islets in the section.

### Quantitative real-time PCR

The total ribonucleic acid (RNA) of the skeletal muscle and adipose tissue was isolated using TRIzol reagent (Invitrogen, Carlsbad, CA), and was used to synthesize complementary deoxyribonucleic acid (cDNA) using the PrimeScript RT reagent kit according to the manufacture’s protocol (TaKaRa Bio, Dalian, China). Then, the amplification reactions were carried out in 96-well reaction plates (Bio-Rad, Hercules, CA) with a 20 μL reaction volume. Glyceraldehyde 3-phosphate dehydrogenase (GAPDH) was used as an endogenous control, and the fold change in the level of the target messenger ribonucleic acid (mRNA) between the control and treatment groups was normalized by the level of GAPDH. The change in gene expression was calculated using 2^−ΔΔCt^ (Xu et al. 2017). Primer sequences for the analysis of insulin receptor (InsR), insulin receptor substrate (IRS)-1, IRS-2 and glucose transporter type 4 (GLUT-4) mRNA in the skeletal muscle and peroxisome proliferator-activated receptor (PPAR)-γ, IRS-1, IRS-2 and GLUT-4 mRNA in the adipose tissue are described in Table 1.

**Table 1.** PCR primers used in all quantitative PCR assays.

Primer: gene	Forward	Reverse
*Gapdh*	5′-CCA AGG TCA TCC ATG ACA AC-3′	5′-TGT CAT ACC AGG AAA TGA GC-3′
*Insr*	5′-GCT GGT GTT TTC CTC ATA GAT TG-3′	5′-CTG TAG GTC AGG TTT GGT TTT TG-3′
*Irs1*	5′-CCT GAC ATT GGA GGT GGG TCT T-3′	5′-GAA TCT TCG GCA GTT GCG GTA T-3′
*Irs2*	5′-TGC GAA CAG CCG TCG GTG AC-3′	5′-GAC CGG TGA CGG CTG AAC GG-3′
*Glut4*	5′-AGC CAG CCT ACG CCA CCA TA-3′	5′-GGA CCC ATA GCA TCC GCA AC-3′
*Pparγ*	5′-CTG GCC TCC CTG ATG AAT AA-3′	5′-GGC GGT CTC CAC TGA GAA TA-3′

### Western blotting

Proteins were extracted from the skeletal muscle and adipose tissue by using lysis buffer. The proteins were then separated using sodium dodecyl sulphate-polyacrylamide gel electrophoresis (SDS-PAGE) (10% or 15%) and electrophoretically transferred into polyvinylidene fluoride membranes. The membranes were probed with anti-InsR (1:20,000; Abcam, Cambridge, UK), anti-IRS-1 (1:500; Abcam, Cambridge, UK), anti-IRS-2 (1:5000; Abcam, Cambridge, UK), anti-GLUT-4 (1:2000; Abcam, Cambridge, UK) and anti-PPAR-γ (1:5000; Abcam, Cambridge, UK) antibodies overnight at 4 °C, and then incubated with a horse radish peroxidase (HRP)-coupled secondary antibody. Detection was performed using a ChemiDoc XRS^+^ (Bio-Rad, Hercules, CA) image analysis system.

### Compound and IR related targets

The targets of LUP were obtained from the Traditional Chinese Medicine Systems Pharmacology Database and Analysis Platform (TCMSP, http://tcmspw.com/tcmsp.-php), Swiss Target Prediction database (http://www.swisstargetprediction.ch/) and BATMAN-TCM (http://bionet.ncpsb.org/batman-tcm/index.php). The genes targets associated with IR were screened using comprehensive database of human genes and gene phenotypes (OMIM, http://www.omim.org/), DrugBank database (https://www.drugbank.ca/) and GeneCards (https://www.genecards.org/).

### Construction of ‘component-target-disease’ network

The LUP-related targets and IR-related targets were linked into a ‘component-target-disease’ network, and the interaction between the proteins were introduced into the STRING database (http://string-db.org/). The above network was visualized using Cytoscape ver. 3.7.1 software (http://www.cytoscape.org/).

### Biological process analysis and pathway analysis

Gene Ontology (GO) functional annotation and the KEGG pathway analysis were performed to explore related biological processes and signalling pathways.

### PPI network analysis and screening of the core targets

Protein–protein interaction (PPI) network analysis was performed using the STRING database. In ‘component-target-disease’ network, there are three key topology parameters in the network including betweenness centrality, closeness centrality and degree, which can be used to screen the more important nodes (Huang et al. 2021). The nodes with degree and betweenness centrality value were two times higher than the median value of all nodes, and the closeness centrality value was greater than the median value of nodes; thus, were chosen as the core targets.

### Molecular docking

The *mol2 format of LUP was downloaded at the TCMSP database, and the 3D structure (*PDB format) format of the targets was obtained from the protein data bank (PDB) database (https://www.rcsb.org/). PyMol software was used to perform dehydration and hydrogenation of the proteins, and docking was performed using the AutoDock Vina software. The results were visualized and analysed.

### Statistical analysis

The data were presented as the mean ± standard deviation (SD). The SPSS 20.0 software (SPSS Inc., Chicago, IL) was used, and the means were assessed using a one-way analysis of variance (ANOVA) and Fisher’s protected least significant difference (LSD) *post hoc* test was used to determine the significant for multiple comparisons. *p* < 0.05 was considered statistically significant.

## Results

### LUP improves body weight loss and decreases the FBG in rats with T2DM

**Figure 1.**
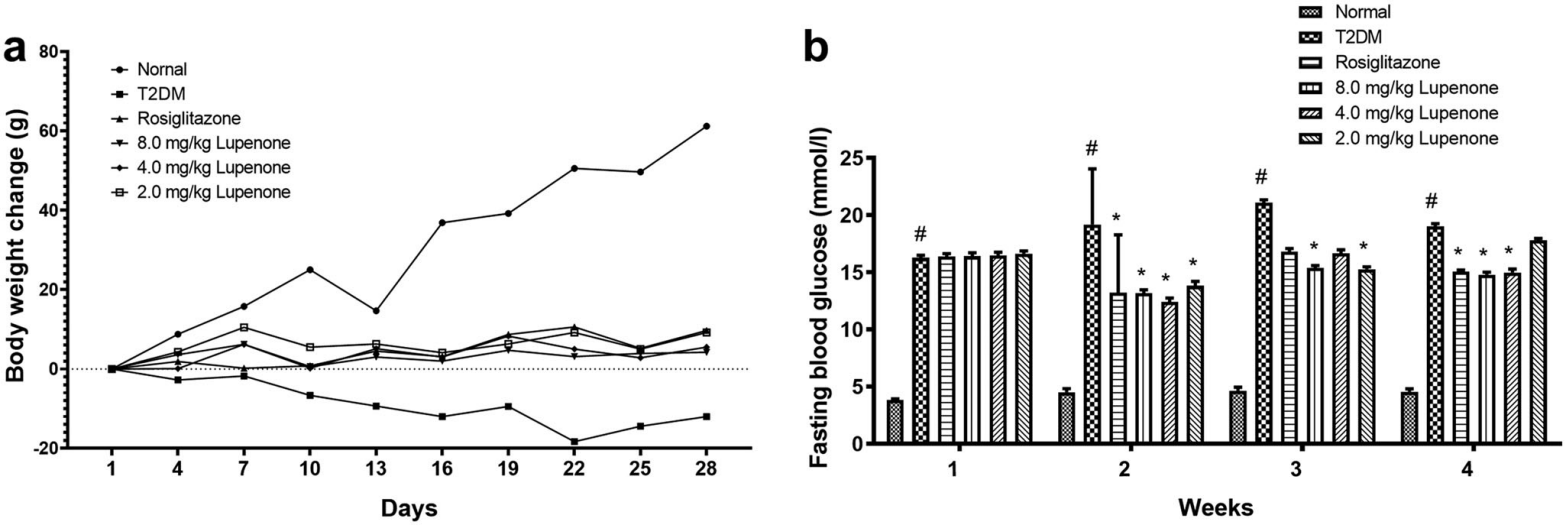
Lupenone prevented the body weight loss (a), decreased the fasting blood glucose level (b) in rats with diabetes. Data presented as mean ± SD. *N* = 9–10 for the change in body weight change and fasting blood glucose. *p< 0.05 vs. the T2DM group, ^#^*p*< 0.05 vs. the normal group. SD: standard deviation.

LUP inhibited the body weight loss induced by STZ. The results are shown in Figure 1(a). After 2 weeks of treatment, the LUP and rosiglitazone significantly decreased the FBG level (*p* < 0.05). After 3 weeks of treatment, LUP (2.0 and 8.0 mg/kg) decreased the FBG level (*p* < 0.05). After 4 weeks of treatment, LUP (4.0 and 8.0 mg/kg) significantly decreased the FBG level of rats (*p* < 0.05). The results showed that LUP has a stable hypoglycaemic effect in rats with T2DM (Figure 1(b)).

### LUP decreases the levels of LEP, HbA1c, MDA and increases the ADPN levels and GSH-PX activity in rats with T2DM

**Figure 2.**
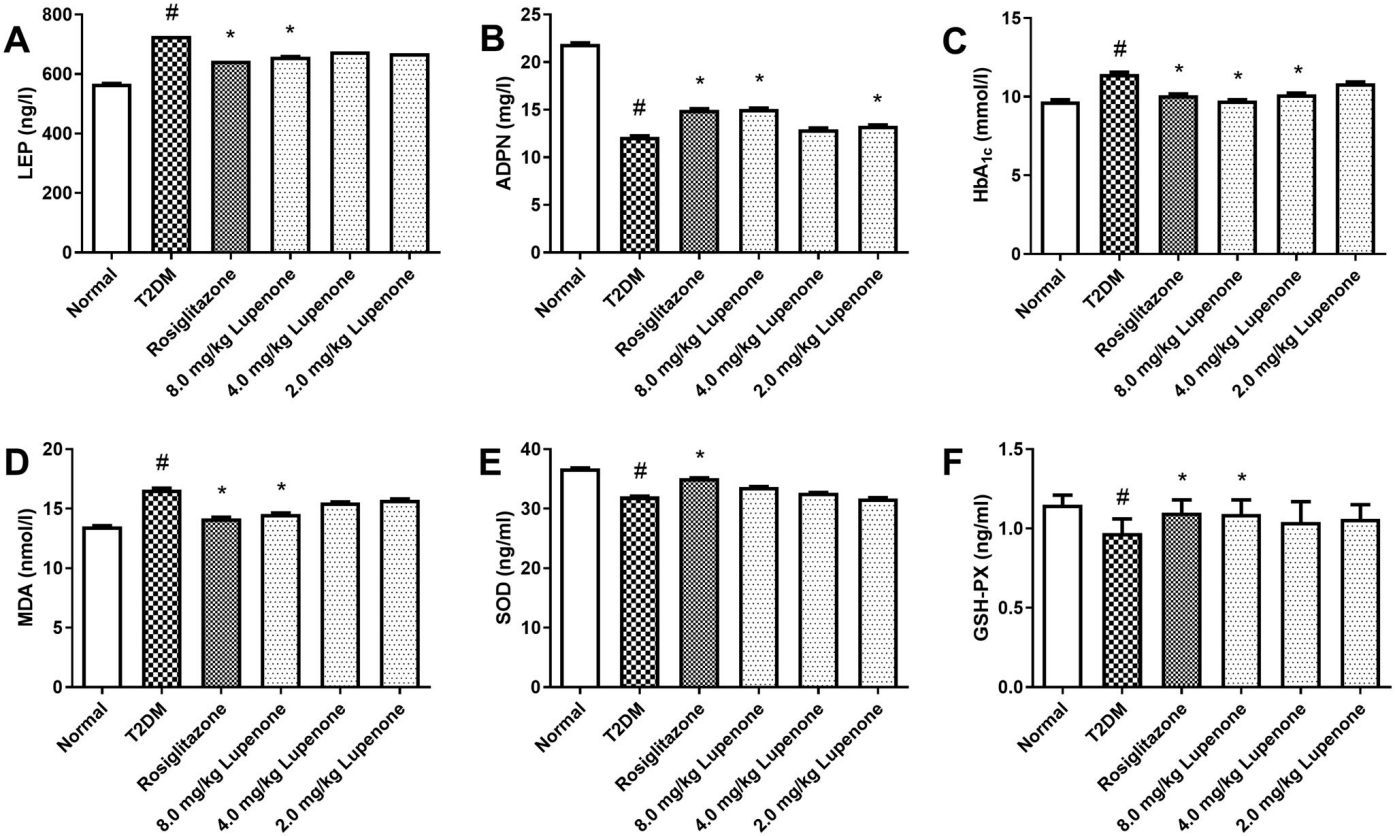
Lupenone decreased the levels of LEP (A) and HbA_1c_ (C), increased the ADPN (B) levels, and reduced the excessive oxidative stress in the serum of rats with diabetes. Lupenone decreased the levels of MDA (D) and reduced GSH-PX (F) activity, but has no significantly effect on the SOD (E) activity. Data are presented as means ± SD. *N* = 9–10 there are nine rats in the normal group and 10 rats in the other groups. *p< 0.05 vs. the T2DM group, ^#^*p*< 0.05 vs. the normal group SD: standard deviation.

After treatment with LUP for 4 weeks, LUP (8.0 mg/kg) and rosiglitazone significantly decreased the levels of LEP in the serum (Figure 2(A)). Additionally, rosiglitazone and LUP (4.0 and 8.0 mg/kg) significantly decreased the serum HbA1c levels in rats with T2DM (Figure 2(C)). Rosiglitazone and LUP (8.0 mg/kg) also significantly decreased the serum MDA levels and increased the serum GSH-PX activity (Figure 2(D,F)). Furthermore, rosiglitazone and LUP (2.0 and 8.0 mg/kg) increased the serum ADPN levels (Figure 2(B)). However, LUP only had a slight effect in the serum SOD activity (Figure 2(E)).

### LUP improves the lipid metabolism disorder in rats with T2DM

**Figure 3.**
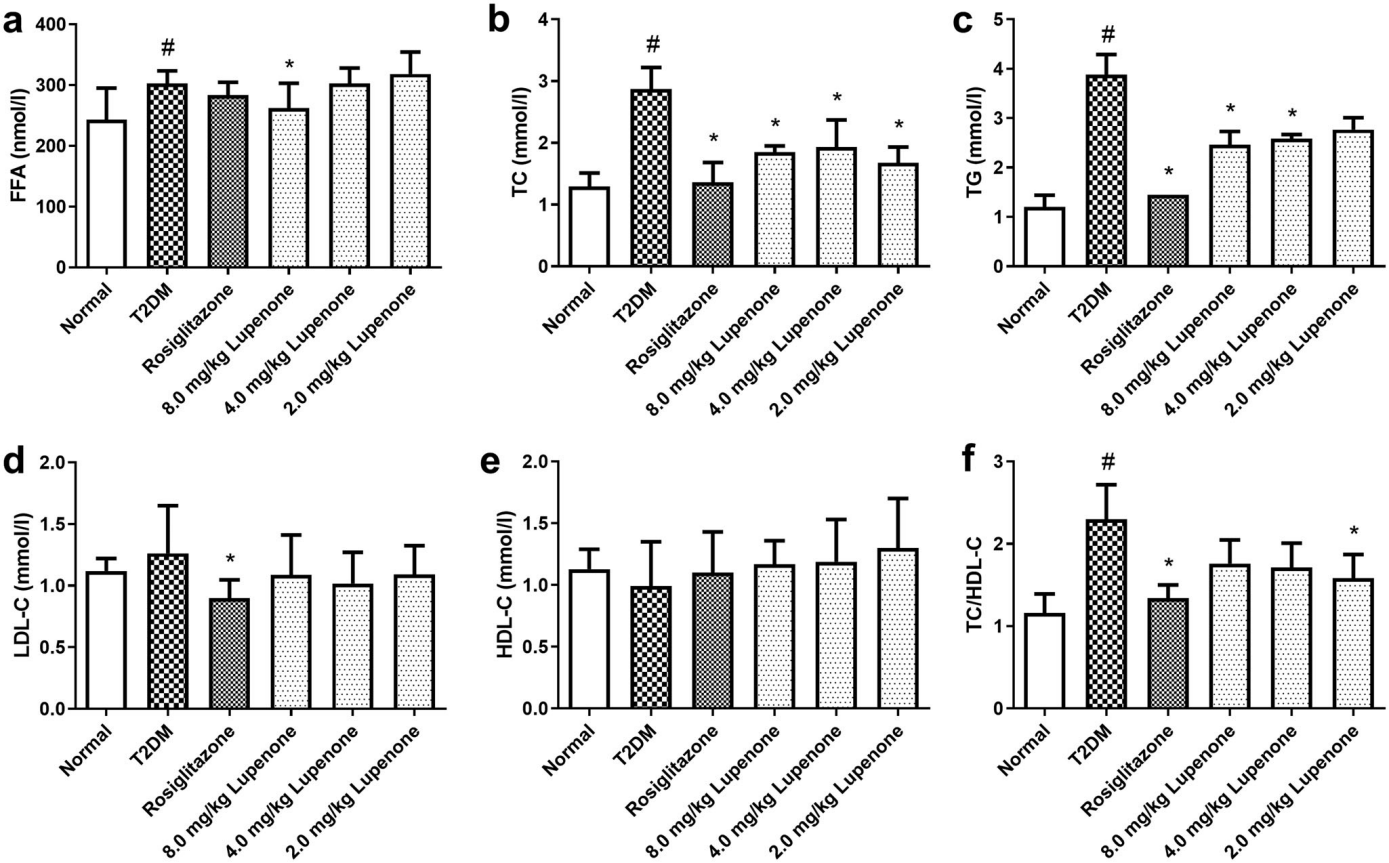
Lupenone improves the lipid metabolic disorder in rats with diabetes. Lupenone decreases the FFA (a), TC (b) and TG (c) levels. Lupenone has no significantly effect on the levels of LDL-C (d) and HDL-C (e), but the 2.0 mg/kg lupenone could decrease the TC/HDL-C (f) ratio. Data are presented as means ± SD. *N* = 9–10 there are nine rats in normal group, whereas and 10 rats in the other groups. *p< 0.05 vs. the T2DM group, ^#^*p*< 0.05 vs. the normal group. SD: standard deviation.

Compared to the normal group, the levels of FFA, TG and TC of the T2DM group were significantly increased, suggesting that the lipid metabolism disorder occurred in the rats with T2DM. LUP (8.0 mg/kg) significantly decreased the levels of FFA, TG and TC in serum (*p* < 0.05). In addition, LUP (2.0 and 4.0 mg/kg) significantly decreased the TC levels and LUP (4.0 mg/kg) reduced the levels of TG in serum (Figure 3(A–C)). Furthermore, rosiglitazone and LUP (2.0 mg/kg) significantly reduced the ratio of TC/HDL-C (Figure 3(F)). However, LUP had no significant effect on LDL-C and HDL-C levels in the serum (Figure 3(D,E)).

### LUP increases the synthesis of hepatic glycogen in rats with T2DM

**Figure 4.**
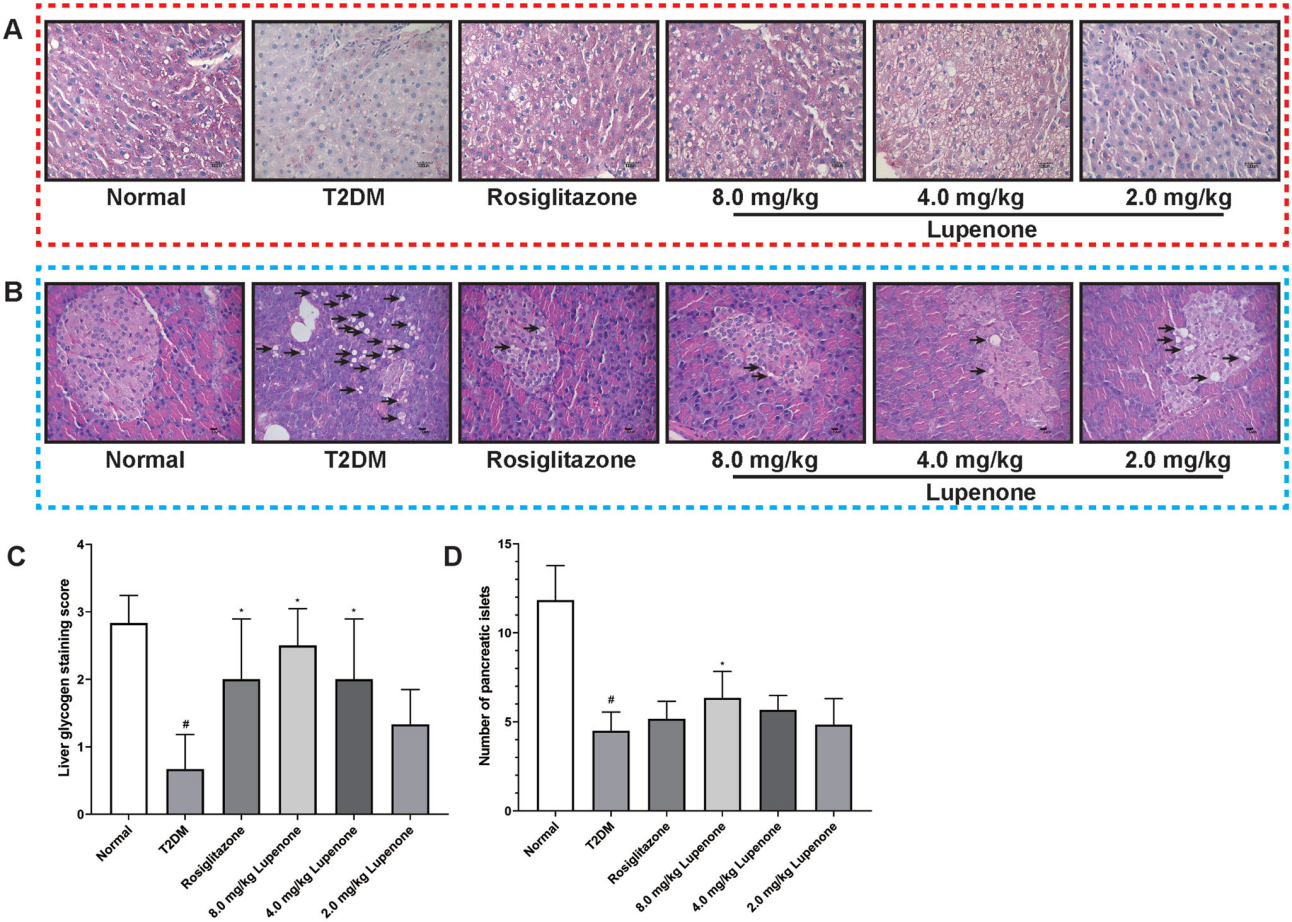
Effects of lupenone on synthesis of hepatic glycogen and pathological changes of the pancreas. (A) Stained reddish dots or threadlike structures in the liver cells are considered positive result; rosiglitazone and lupenone increased the synthesis of the hepatic glycogen in rats with diabetes (×100 magnification; *n* = 6). (B) Acinar cell steatosis (short arrows) and small irregular-shaped islet are observed in the pancreas, rosiglitazone and lupenone protected the structure of the exocrine part of the pancreatic tissues and decreased fat degeneration (×400 magnification; *n* = 6). (C) Quantitative analysis of the synthesis of hepatic glycogen in rats with T2DM. (D) The number of islets.

The liver cell morphology of T2DM group was complete, but the interstitial had hyperplasia. The cell gaps of the hepatocytes were large, cells were irregularly arranged, the intercellular and intracellular PAS staining was low, and the relative homogeneity was poor. However, in the normal, rosiglitazone and the LUP (8.0 mg/kg) groups, the structure of the hepatocytes was tight and neat with no cell gaps. Furthermore, the intercellular and the intracellular PAS staining were diffusely coloured and had good homogeneity. Compared to the T2DM group, the interstitial hyperplasia was less, cells gap was smaller, the cells were arranged regularly, and PAS-positive cells were significantly increased in the LUP (2.0 and 4.0 mg/kg) group. The rosiglitazone and LUP could increase the synthesis of hepatic glycogen in rats with T2DM (Figure 4(A,C)).

### LUP improves the pathological changes of the pancreas in rats with T2DM

The structure of the exocrine part of the pancreatic tissue in rosiglitazone and LUP (2.0, 4.0 and 8.0 mg/kg) group was normal, the endocrine part of the pancreatic tissue had a small amount of fatty degeneration and no obvious inflammatory cell infiltration was observed (Figure 4(B)), and 8.0 mg/kg LUP could significantly inhibit the number of islets reduction (Figure 4(D)).

### LUP increases the mRNA and protein expression of InsR, IRS-1, IRS-2, GLUT-4 in skeletal muscle and PPAR-γ, IRS-1, IRS-2, GLUT-4 in the adipose tissue of T2DM rats with T2DM

**Figure 5.**
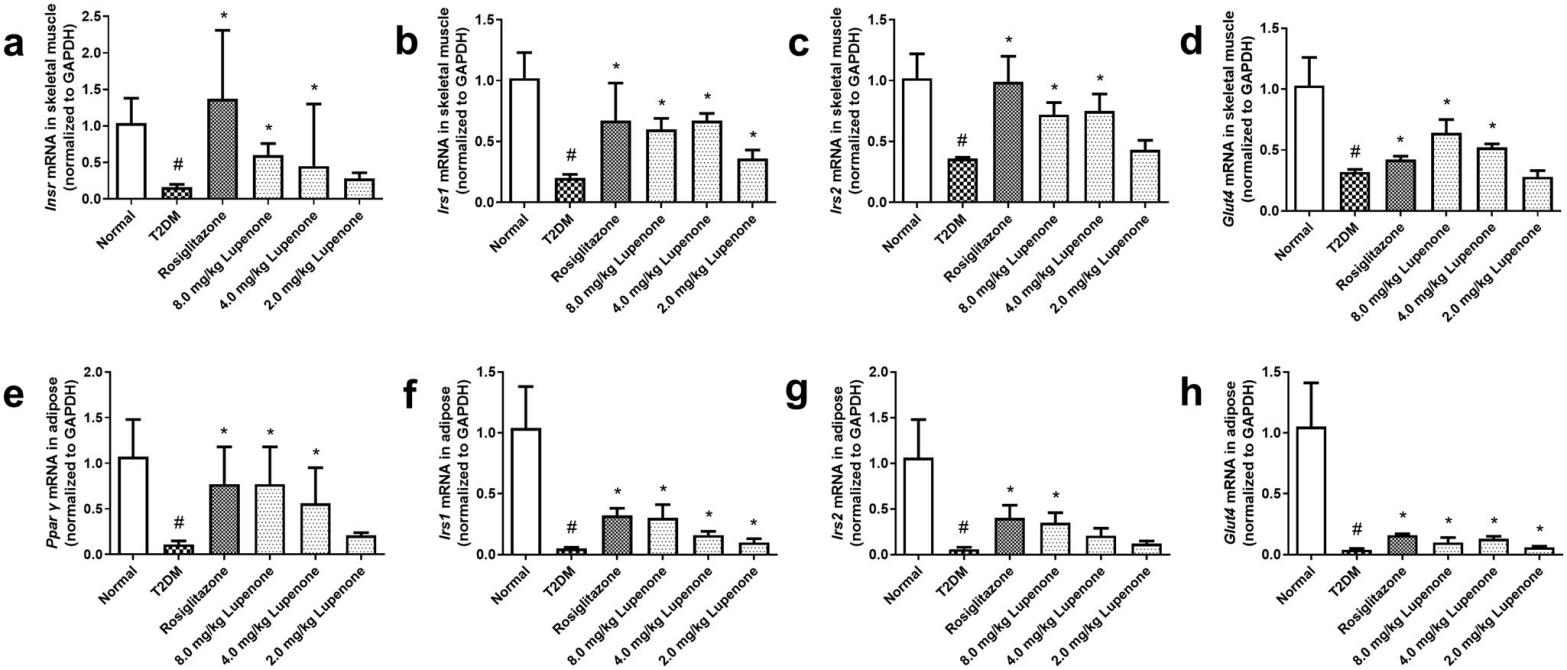
Lupenone increased the mRNA expression in the skeletal muscle and adipose tissue related to insulin resistance of rats with diabetes. Lupenone increased the mRNA expression of InsR (a), IRS-1 (b), IRS-2 (c) and GLUT-4 (d) in the skeletal muscle and PPAR-γ (e), IRS-1 (f), IRS-2 (g) and GLUT-4 (h) in the adipose tissue of rats with diabetes. Data are presented as means ± SD. *n* = 6. *p< 0.05 vs. the T2DM group, ^#^*p*< 0.05 vs. the normal group; SD: standard deviation.

The mRNA expression of InsR, IRS-1, IRS-2 and GLUT-4 in the skeletal muscle and PPAR-γ, IRS-1, IRS-2 and GLUT-4 in the adipose of rats with T2DM is decreased (Figure 5(A–H)). LUP (4.0 and 8.0 mg/kg) and rosiglitazone significantly increased the expression of InsR, IRS-1, IRS-2 and GLUT-4 mRNA in the skeletal muscle (*p* < 0.05) and PPAR-γ, IRS-1, IRS-2 and GLUT-4 mRNA in the adipose tissue (*p* < 0.05). In addition, LUP (2.0 mg/kg) significantly increased the expression of IRS-1 mRNA in the skeletal muscle (*p* < 0.05). In addition, LUP (2.0 and 4.0 mg/kg) significantly increased the expression of IRS-1 and GLUT-4 mRNA in the adipose tissue, and LUP (4.0 mg/kg) significantly increased the expression of PPAR-γ mRNA in the adipose tissue (*p* < 0.05).

**Figure 6.**
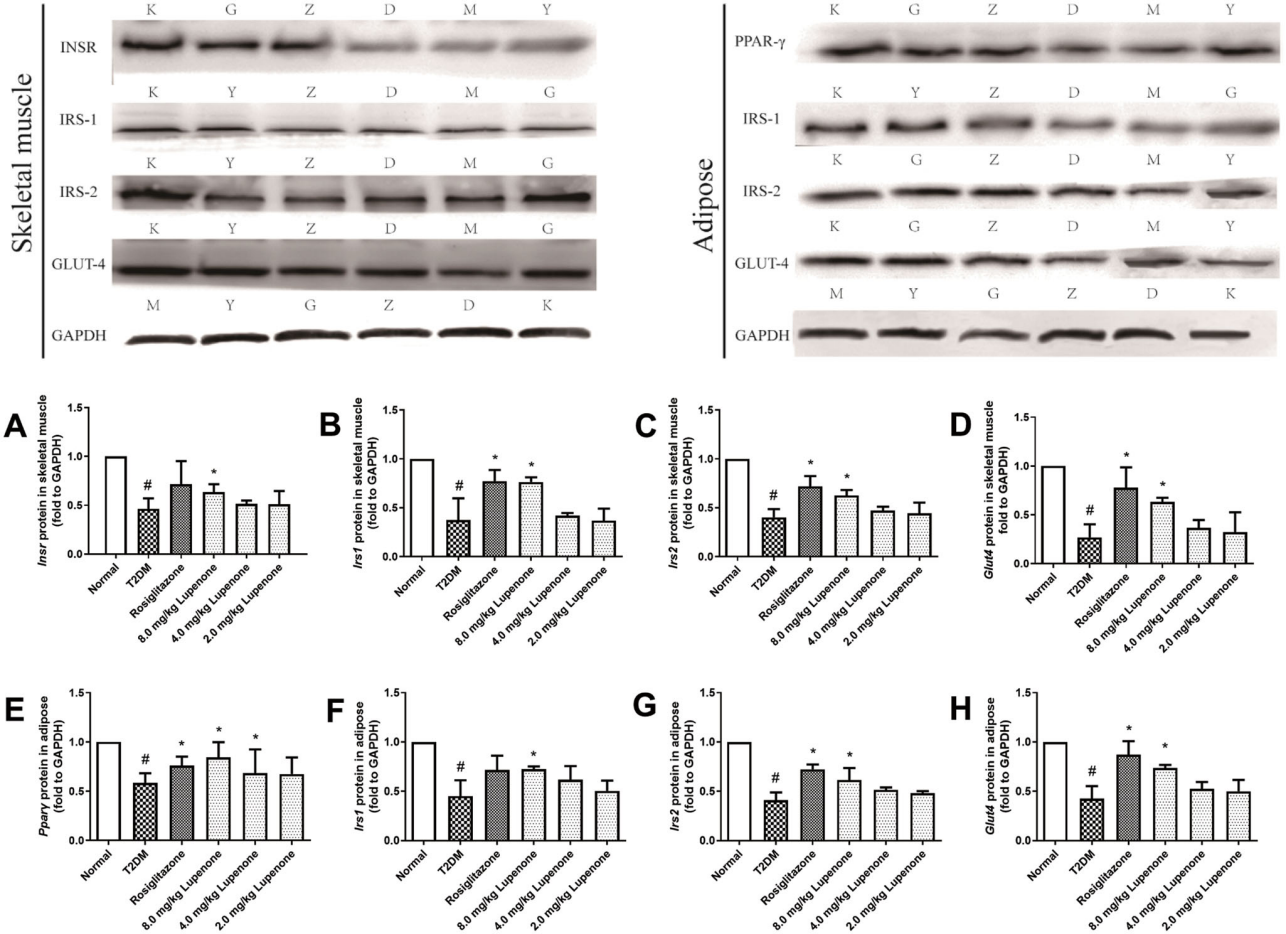
Lupenone increased the protein expression in the skeletal muscle and adipose tissue related to insulin resistance of rats with diabetes. Lupenone increases the protein expression of InsR (A), IRS-1 (B), IRS-2 (C) and GLUT-4 (D) in the skeletal muscle and PPAR-γ (E), IRS-1 (F), IRS-2 (G) and GLUT-4 (H) in the adipose tissue of rats with diabetes. Data are presented as means ± SD. *n* = 6. *p< 0.05 vs. the T2DM group, ^#^*p*< 0.05 vs. the normal group. SD: standard deviation. K: normal, M: T2DM, Y: rosiglitazone, D: 2.0 mg/kg lupenone, Z: 4.0 mg/kg lupenone, G: 8.0 mg/kg lupenone.

The protein expression of InsR, IRS-1, IRS-2 and GLUT-4 in the skeletal muscle and PPAR-γ, IRS-1, IRS-2 and GLUT-4 in the adipose tissue of rats with diabetes is significantly decreased (Figure 6(A–H)). Rosiglitazone significantly increased the protein expression of IRS-1, IRS-2 and GLUT-4 in the skeletal muscle and PPAR-γ, IRS-2 and GLUT-4 in the adipose tissue (*p* < 0.05). LUP (8.0 mg/kg) significantly increased the protein expression of InsR, IRS-1, IRS-2 and GLUT-4 in the skeletal muscle and PPAR-γ, IRS-1, IRS-2 and GLUT-4 in the adipose tissue (*p* < 0.05). In addition, LUP (4.0 mg/kg) increased the protein expression of PPAR-γ in the adipose tissue (*p* < 0.05).

### Network analysis

**Figure 7.**
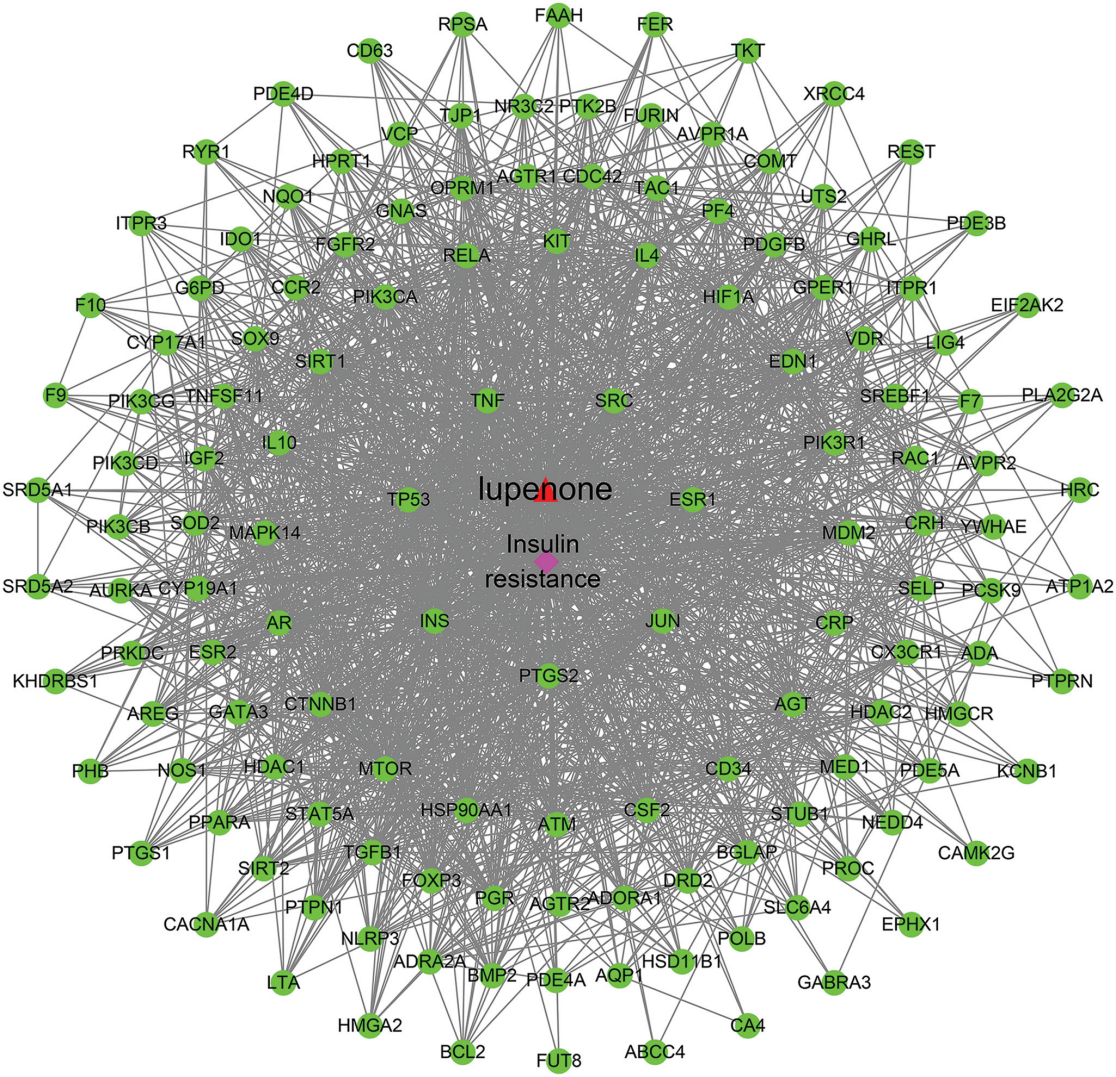
‘Lupenone-target-disease’ interaction network. The red triangle is the chemical component, the pink diamond is the disease, and the green circle is the common target genes of the component and the disease, and the line represents each side, indicating the interaction between the components and the targets.

An interactive network of LUP to improve IR is constructed and visualized using different colour and shape patterns (Figure 7). Among these, the red triangle is the chemical component, the pink diamond is the disease and the green circle is the common target genes of the component and the disease.

### GO functional annotation and KEGG pathway analysis

**Figure 8.**
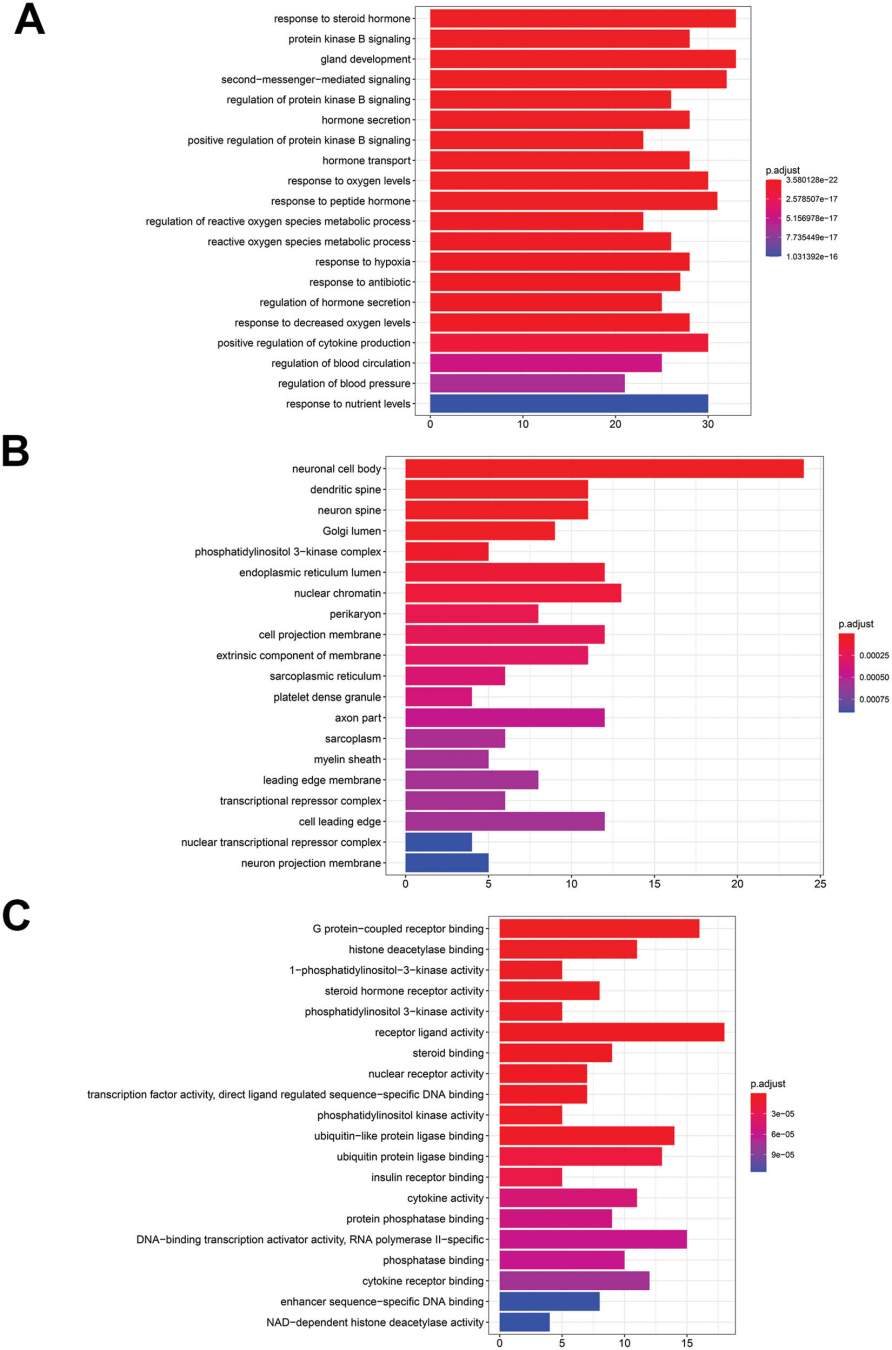
GO enrichment analysis of target proteins. GO enrichment analysis include biological process (A), cellular component (B) and molecular function (C).

GO functional annotation was performed. Potential targets (139) were used to perform the biological functional enrichment using the R packages ggplot2 and cluster profiler. A total of 2724 biological processes with a total *p* value of ≤0.01 were enriched. A biological process is most strongly correlated with response to steroid hormone, protein kinase B signalling and gland development, etc. (Figure 8(A)); In the cellular component, there are 110 biological processes with *p* value ≤0.01, including the neuronal cell body, dendritic spine, neuron spine and Golgi lumen, etc. (Figure 8(B)); In the molecular function, there are 150 biological processes with *p* value ≤0.01, among which G protein-coupled receptor binding, histone deacetylase binding and 1-phosphatidylinositol-3-kinase activity are the strongest (Figure 8(C)).

**Figure 9.**
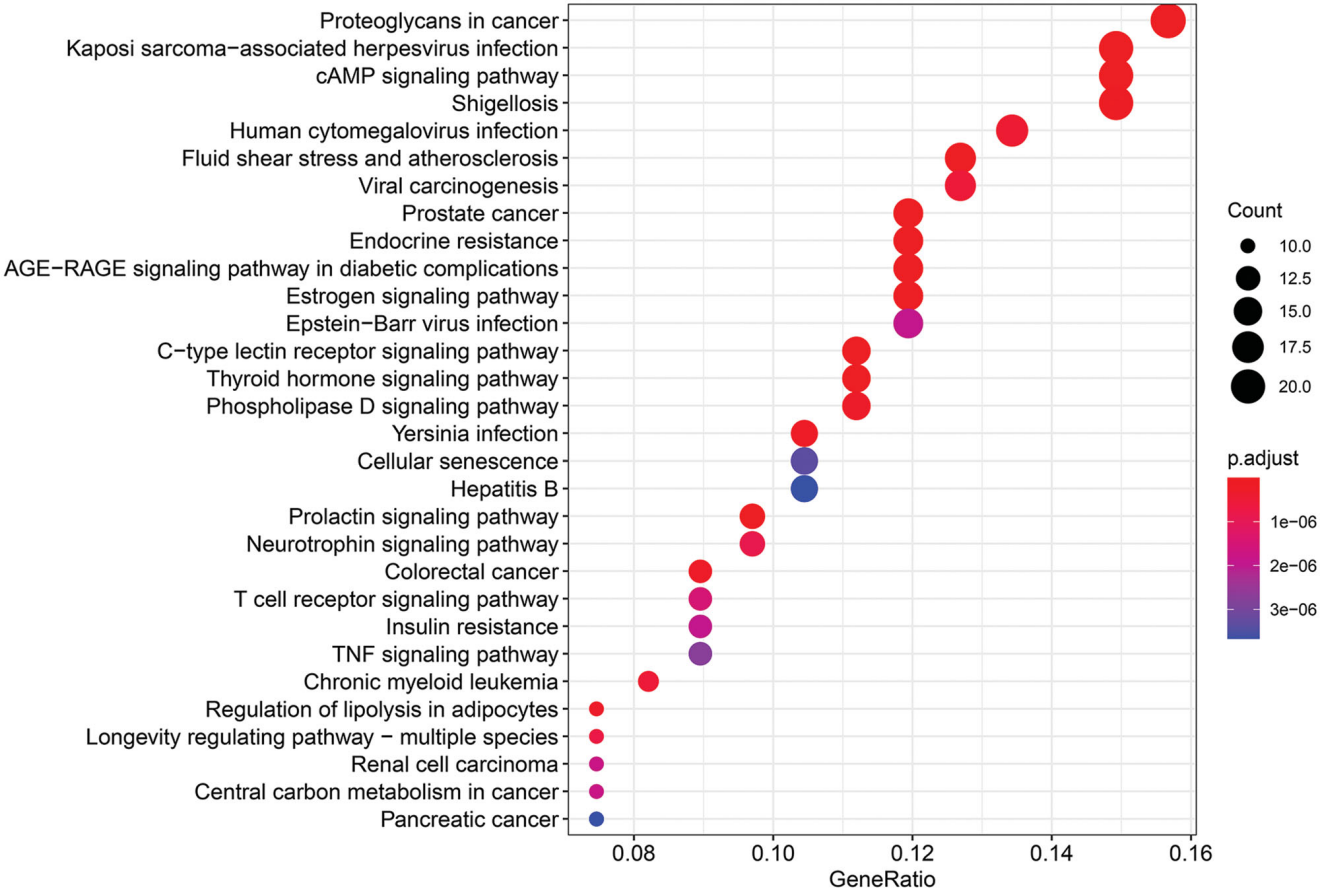
The KEGG pathway analysis of the targets.

**Figure 10.**
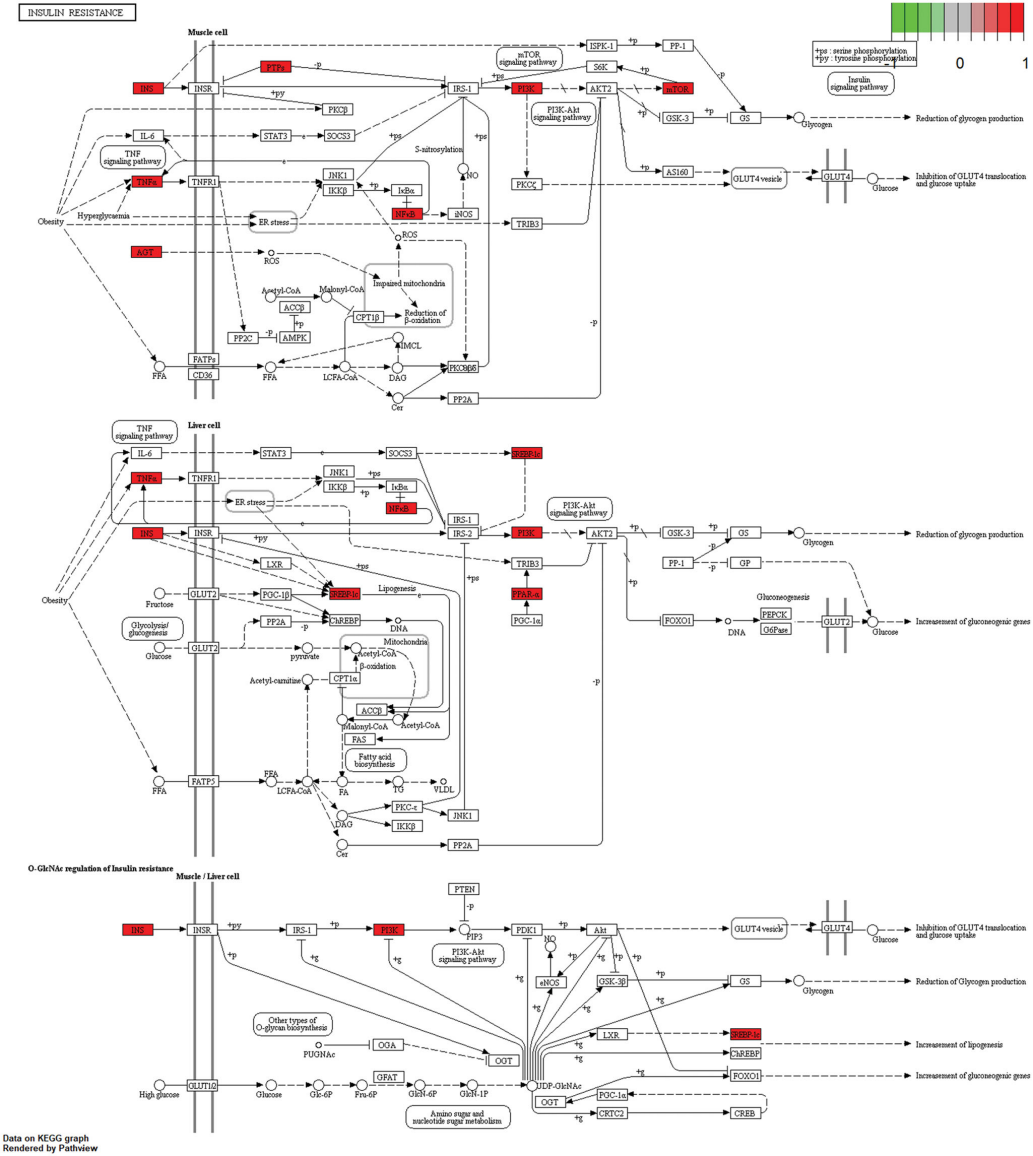
Insulin resistance. The direct targets of lupenone on insulin resistance are shown. The red displays the key targets.

A total of 164 pathways were screened using the KEGG enrichment analysis (*p*≤ 0.01), and some of the signal pathways are shown in Figure 9. The LUP might improve IR by acting on prostate cancer, endocrine resistance, advanced glycation end products/receptor for advanced glycation end products (AGE-RAGE) signalling pathway in diabetic complications, proteoglycans in cancer, and kaposi sarcoma-associated herpesvirus infection. The targets of the effect of LUP on IR are shown in Figure 10.

### PPI network analysis and the core targets results

**Figure 11.**
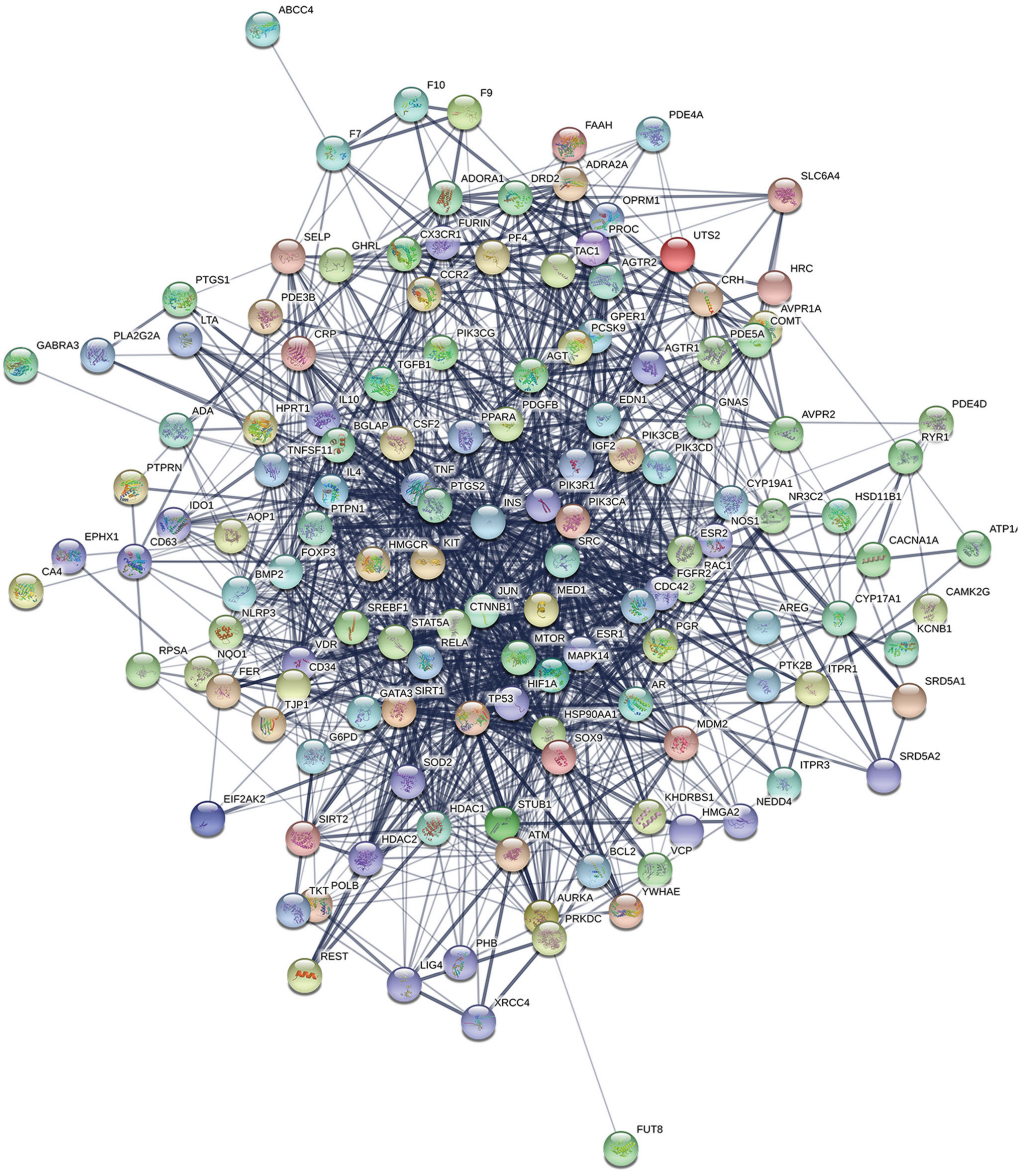
Interaction network of targets, PPI enrichment *p* value: *p*< 1.0e–16.

The targets are introduced into the STRING database, and the specie was ‘*Homo sapiens*’, and then the interaction network of the protein targets was obtained, as shown in Figure 11. There was a total of 139 nodes and 1357 edges in the network. Additionally, the results of the topological analysis in the network showed that the median value of the betweenness centrality, closeness centrality and degree was 0.000577, 0.534351 and 18, respectively. According to the screening criteria, 24 hub targets of LUP were obtained. The results are shown in Table 2.

**Table 2.** Topological analysis of core targets in ‘lupenone -target-disease’ network.

Gene name	Betweenness centrality	Closeness centrality	Degree
INS	0.068121	0.756757	95
TP53	0.02875	0.679612	74
TNF	0.023754	0.669856	71
SRC	0.022054	0.660377	68
ESR1	0.013141	0.633484	59
JUN	0.009126	0.625	56
PTGS2	0.008946	0.611354	51
HSP90AA1	0.01071	0.608696	50
CTNNB1	0.009412	0.606061	49
MTOR	0.008296	0.606061	49
AR	0.007686	0.598291	46
MAPK14	0.005711	0.595745	45
IL10	0.006232	0.59322	44
SIRT1	0.005683	0.59322	44
PIK3CA	0.005147	0.588235	42
IL4	0.005161	0.585774	41
KIT	0.004878	0.585774	41
RELA	0.003582	0.585774	41
HIF1A	0.004726	0.583333	40
EDN1	0.004736	0.578512	38
PIK3R1	0.004544	0.578512	38
MDM2	0.004322	0.578512	38
AGT	0.006154	0.576132	37
CRP	0.005963	0.576132	37

### Molecular docking results

**Figure 12.**
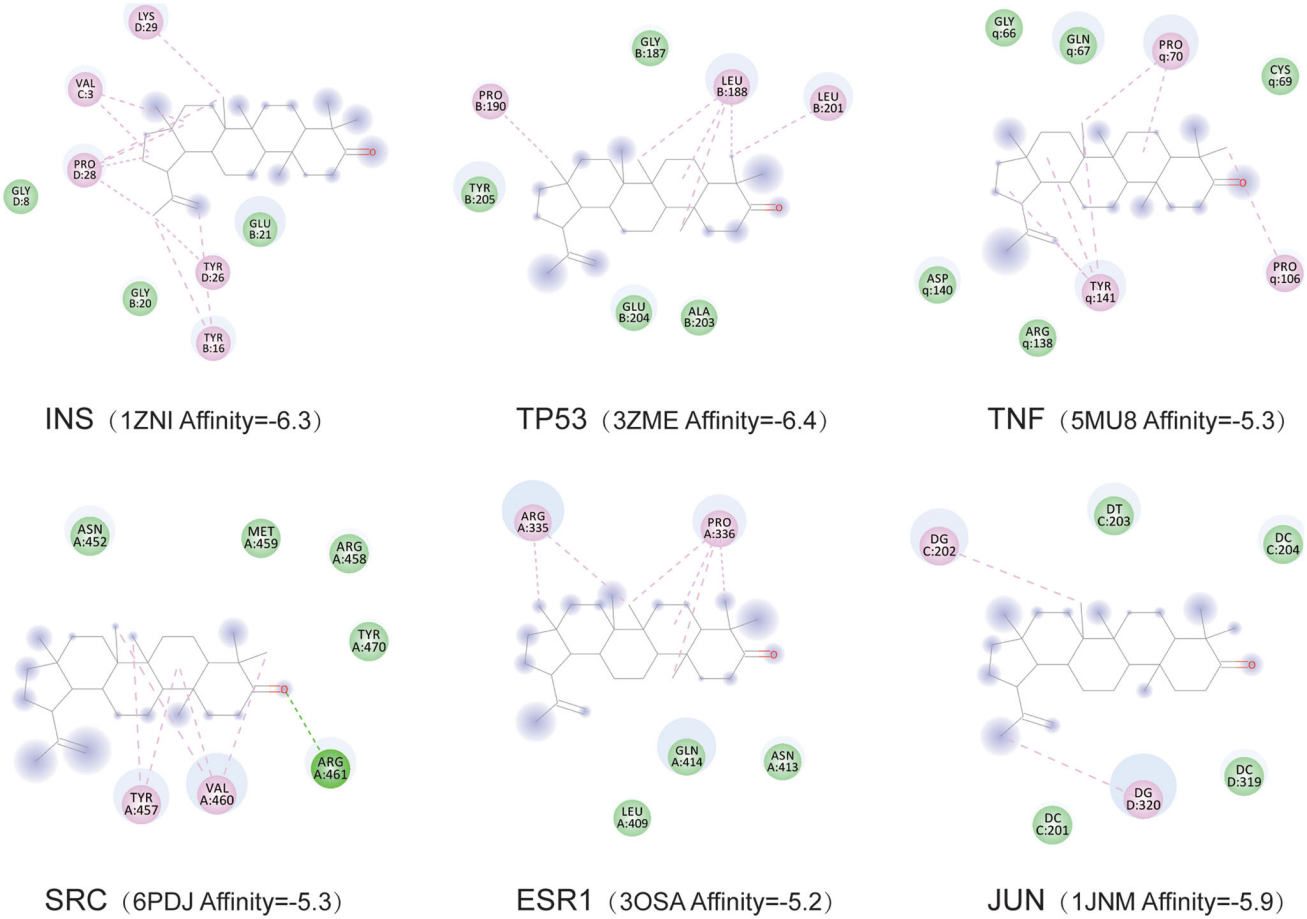
Molecular docking results of lupenone and the corresponding protein of the gene targets.

The conformation with the highest score (lowest affinity value) was selected, and the docking conformation was visualized using the PyMol software. The results showed that the LUP had good affinity with the proteins corresponding to the genes including INS, tumour protein p53 (TP53), tumour necrosis factor (TNF), SRC.oestrogen receptor 1 (ESR1) and Jun Proto-Oncogene, AP-1 Transcription Factor Subunit (JUN) (Figure 12). These six genes were the most relevant genes for LUP to improve IR according to the network analysis.

## Discussion

In this study, we found that LUP could inhibit the body weight loss and decrease the FBG levels. Furthermore, LUP improved the lipid metabolic disorders and reduced the excessive oxidative stress in rats with T2DM. Moreover, LUP promoted the synthesis of hepatic glycogen and improved the pathological changes of the pancreas. LUP could improve the IR via regulation of gene and protein expression in rats with T2DM. In addition, network pharmacology analysis revealed that LUP improves IR by multiple targets and signal pathways. These results indicate that LUP has the potential to be developed as a new drug for the treatment of T2DM.

Rats with induced HFD/STZ T2DM are often accompanied by weight loss (Sharma et al. 2011; Wei et al. 2017), but there are conflicting reports on the changes in body weight of rats with T2DM after drug treatment (Gheibi et al. 2017). In this study, LUP could inhibit the body weight loss of rats T2DM. In addition, LUP decreased the LEP level, which is closely related to the appetite, body weight, neuroendocrine functions and glycaemia (Coppari and Bjørbaek 2012). The above results suggested that LUP has a good effect on weight maintenance of rats with T2DM. As we know, HbA1c was formally included in the diagnostic index of T2DM by the American Diabetes Association (ADA) in 2010. LUP could reduce the FBG and HbA1c levels in rats with T2DM, and the result is consistent with our previous study (Xu et al. 2014). Furthermore, the IR is closely related to the hepatic glycogen metabolism disorders, LUP could increase the synthesis of the hepatic glycogen; thus, LUP might improve the IR by regulating gluconeogenesis in rats with T2DM.

Lipid metabolism disorders are closely related to the cardiovascular diseases and T2DM (Loza-Rodríguez et al. 2020), and TC/HDL-C ratio.TC/HDL-C ratio is a reasonable blood lipid index in predicting the cardiovascular risk of patients with T2DM. Our results showed that LUP significantly decreases the levels of TC, TG and TC/HDL-C ratio, which indicates that LUP can improve the lipid metabolism disorders and may reduce cardiovascular risk patients with T2DM.

The oxidative stress caused by obesity, hyperglycaemia, FFA and inflammation could lead to the islet β cell damage (Halim and Halim 2019). As reported, long-term exposure to high concentrations of FFA will damage the islet β cell function that reduces the secretion of insulin, increased the gluconeogenesis, and eventually lead to IR (Zhande et al. 2002). Moreover, ADPN can increase the muscle and liver sensitivity to insulin by increasing the FFA oxidation (Wang et al. 2009); hence, ADPN level is an important marker for improvement of IR (Yukihiro et al. 2004). LUP could decrease FFA levels and increase ADPN secretion in rats with T2DM. This study indicates that LUP could improve the IR in rats with T2DM. Furthermore, levels of SOD, MDA and GSH-PX are closely related to the islet β cells damage (Coskun et al. 2005; Sun et al. 2007). LUP increased the GSH-PX activity and decreased MDA levels in rats with T2DM. Interestingly, LUP also inhibited the mild inflammatory response in the pancreatic tissue. Taken together, LUP might protect the patient with T2DM via inhibiting the inflammation, and the result is consistent with our previous study (Xu et al. 2020). InsR/phosphatidylinositol-3 kinase/glucose transporter (PI3K/GLUT-4) signalling pathway plays an important role in improving IR in the skeletal muscle and adipose tissue (Liu et al. 2012). This study found that LUP could increase the gene and protein expression of InsR in the skeletal muscle and GLUT-4 in the skeletal muscle and adipose tissue. Network pharmacology analysis results showed that LUP can improve IR by acting on PI3K (Figure 10). These results indicate that LUP might increase the sensitivity of the skeletal muscle and adipose tissue to insulin, at least partly, by activating the InsR/PI3K/GLUT-4 signalling pathway. According to the report, the expression of IRS-1 in the skeletal muscle and adipose tissue of patients with T2DM is significantly decreased (Ducluzeau et al. 2001; Hammarstedt et al. 2003). Both IRS-1 and IRS-2 function in the peripheral carbohydrate metabolism, and IRS-2 has the major role in the β-cell development and compensation for peripheral IR (Withers et al. 1999). In the present study, LUP up-regulated the gene and protein expression of IRS-1 and IRS-2 in the skeletal muscle and adipose tissue, suggesting that LUP might improve the IR via acting on the family of insulin substrates in T2DM. In addition, peroxisome proliferator-activated receptor gamma (PPAR-γ) is known to play a major role in the improvement of insulin sensitivity through stimulating differentiation of small adipocytes from preadipocyte (Kadowaki et al. 2003). Here, LUP might improve the IR by up-regulating the expression of PPAR-γ. Therefore, the above results showed that LUP improves the skeletal muscle and adipose tissue sensitivity to insulin through increasing the gene and protein transcription related to IR in T2DM.

Combining the drug-target network with the biological system network can provide new approaches and strategies for new drug development (Hopkins 2007). Network pharmacological studies have found that LUP improves IR through multiple targets and multiple pathways. The biological process is mainly related to the regulation of cell proliferation, signal transduction, metabolism and cell differentiation. The results suggested that LUP could improve IR by regulating multiple biological processes. The KEGG pathway analysis also showed that LUP improves IR involving multiple signalling pathways, like AGE-RAGE signalling pathway in diabetic complications, IR and type II diabetes mellitus. AGEs play a significant role in the pathogenesis of multiple diabetic complications (Sergi et al. 2021). These results indicated that LUP might be used for the prevention and treatment of T2DM associated complications. Of course, many signalling pathways related to cancer, tumour, atherosclerosis and infection were predicted, indicating that LUP might have various modes of pharmacological activities, which deserves further research.

## Conclusions

LUP could improve the lipid metabolism disorder and alleviate the excessive oxidative stress in T2DM. Furthermore, as expected, LUP improved the IR via increasing the sensitivity of the skeletal muscle and adipose tissue to insulin by regulating related gene and protein expression in T2DM. Our preliminary results indicate that LUP has the potential to be developed as a new drug for the treatment of T2DM.

## Authors contributions

Feng Xu and Mei Zhang contributed equally to this work. Xiangpei Wang, Feng Xu, and Hongmei Wu contributed to the study conception and design, acquisition, analysis and interpretation of data, and writing of the article. Mei Zhang, Yuanmin Wang and Ye Yang contributed to the acquisition and interpretation of data and to the final critical revision of the article. All authors read and approved the manuscript.
